# Supplementary material for: The Impact of Point-of-Care Ultrasound on the Diagnosis and Management of Small Bowel Obstruction in the Emergency Department: A Retrospective Observational Single-Center Study
Source: Medicina (Kaunas). 2024 Dec 4;60(12):2006. doi: 10.3390/medicina60122006 (PMC11727861; doi:10.3390/medicina60122006)
Supplement: Supplementary file 1 [file medicina-60-02006-s001.zip › medicina-3295447-supplementary.pdf]

Supplementary Material

Figure S1. Relation between the time to diagnosis (y-axis) and NEWS classes (x-axis). Group 0: non-POCUS group; Group 1: POCUS group.

Figure S1

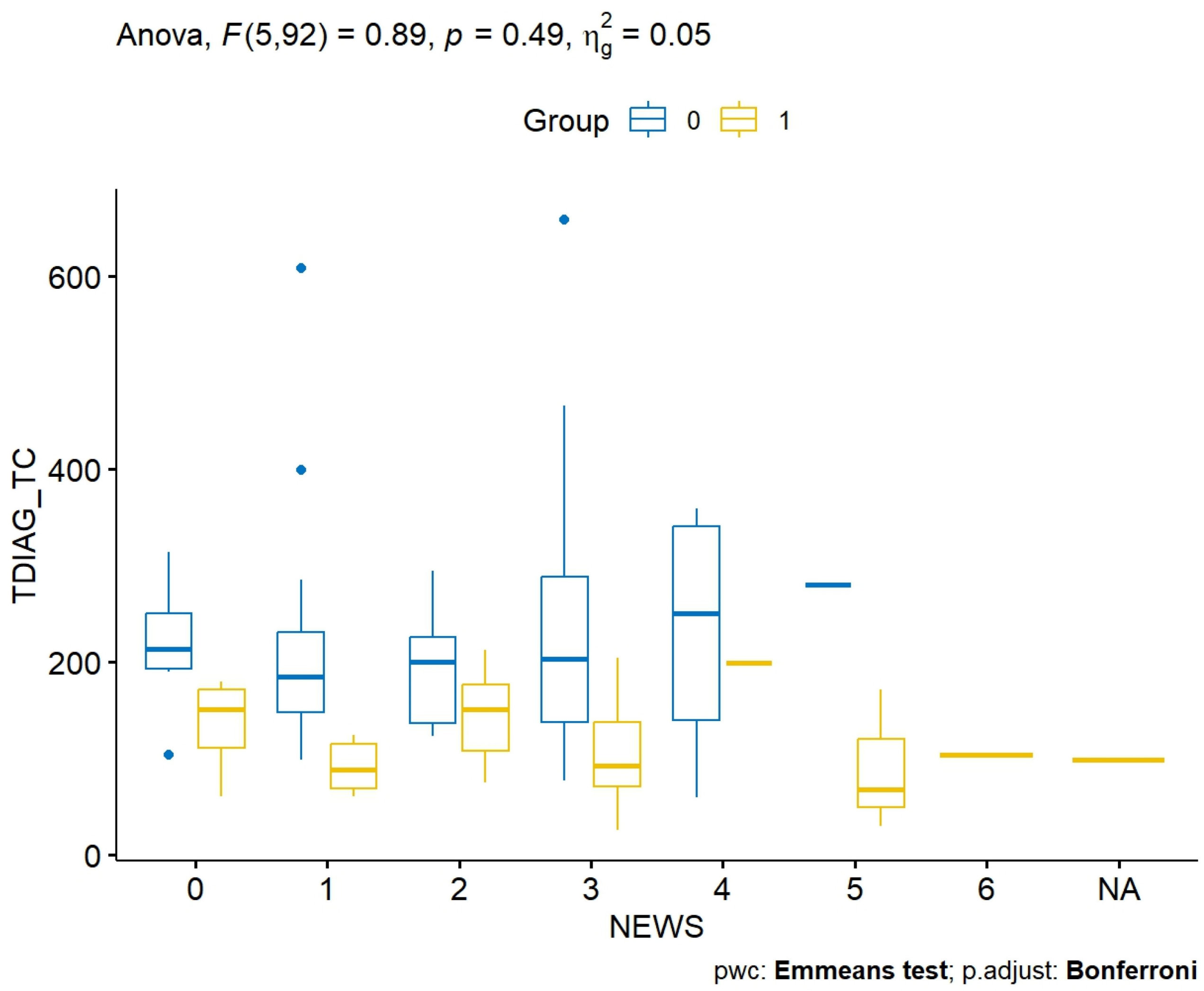

Anova, F(5,92) = 0.89, p = 0.49, η²g = 0.05
Group 0 1
TDIAG_TC
600
400
200
0
0 1 2 3 4 5 6 NA
NEWS
pwc: **Emmeans test**; p.adjust: **Bonferroni**
